# Progress in Bacteriology

**Published:** 1902-12-13

**Authors:** 


### Progress in Bacteriology.

**Bactericidal Effects of Human Blood.**—Wright and Windsor[1] have made an exhaustive investigation into this question. Their conclusions are as follows: The typhoid bacillus and cholera vibrio are readily killed by normal serum, but the various strains of staphylococci, B. pestis, micrococcus melitensis, and probably streptococci and B. diphtheriæ are not affected by the same. A point of great interest is that if sterilised cultures of B. typhosus or the cholera vibrio be introduced into the serum, the bactericidal power is greatly augmented, a fact that throws some light upon the beneficial effects of anti-cholera and anti-typhoid inoculations. In addition to the bactericidal power of serum, the white corpuscles have themselves bactericidal power, owing to their phagocytic action. Heine[2] has lately shown that the red blood corpuscles have, too, a low bactericidal action; an action much slower than that of the serum, but capable in from three to four days of setting up degenerative changes in bacteria.

**Leprosy.**—Van Houtum[3] records the fact that he has successfully cultivated the leprosy bacillus in artificial media. Excising a small nodule of leprous tissue, he tried various media without success, but finally found one suitable, viz., fish broth or a mixture of fish and peptone broth. With difficulty subcultures were made in agar with or without glycerin or glucose. The bacilli differed from those described by Hansen both in staining reaction and size, but they gave a specific reaction (Pfeiffer-Bordet) with leprous serum and not with serum of healthy persons.

**Trypanosome in Human Blood.**—Trypanosomes occuring in the blood of animals are not uncommon and are associated with the diseases in horses, mules, and cattle known as Surra, Nogana, Mal de Caderas, and Dourine. Dutton[4] records a case in which a trypanosome was found in the blood of an Englishman resident on the West African Coast. His symptoms were, wasting and weakness of the lower limbs, irregular relapsing fever, œdema about the eyes, injection of skin and conjunctiva, enlargement and tenderness of spleen, frequent pulse and respiration. The parasite as seen in the fresh blood is a minute worm-like body, with a long whip-shaped flagellum which imparts a peculiar screw-like movement. Blood films stained by Romanowsky's method show the parasite to be from 18–25$\mu$ in length by 2–2·8 in width. It possesses a nucleus, a centrosome and an undulating membrane. The only previous instance of a trypanosome being found in the blood is that recorded in 1888 by Neporie, but this appears to have been a different variety. In an appendix to the report on the Yellow Fever Expedition it is mentioned that trypanosomes were found in the blood of a mosquito that had been placed in a cage with a bat, but although during the expedition some eighty mosquitoes were dissected, this was the only time that trypanosomes were found.

**Infection by B. Coli.**—Paratyphoid fever. Klein[5] has isolated from ice cream a bacillus of the coli group which differs entirely from the bacillus of Gaertner, but which is rapidly fatal to rodents, producing symptoms of gastro enteritis and peritonitis. The ice cream from which the cultures were obtained had given rise to an outbreak of gastro enteritis, and when injected in small quantities (½ c.c.) into guinea-pigs set up peritonitis and fatal septicæmia. Other samples were taken from shops supplied by the same firm, and all these proved to be toxic, and the poisonous condition persisted until improved hygienic measures of manufacture and storage were adopted. As the materials employed were found to be harmless, the trouble must have arisen from the filthy methods of storage and preparation. The materials for making the ice cream are mixed in large quantities, and frozen from day to day as required, and it is in this "stock" that the putrefactive changes take place. This variety of colon bacillus could not be derived from the air, for recent exhaustive examinations of London air failed to demonstrate the presence of coli organisms, the chief varieties being micrococci, sarcinæ, bacilli, streptothrices, and yeasts. Vaughan[6] has recently succeeded in isolating the toxin of the bacillus coli. The toxin is contained within the germ cell and is but very slightly affected by heat or acid. It may be obtained and preserved in the dried state by digesting the bacilli with 2 per cent. hydrochloric acid and ½ per cent. pepsin. The opinion is gradually gaining ground that although the bacillus typhosus is a specific organism, and it alone can give rise to true typhoid fever, and give an agglutinating reaction with the serum of the typhoid patient, yet there are other members of the colon group which can excite a disease clinically resembling enteric, and differing only in the fact that the blood is not capable of clumping the cultures of the Eberth-Gaffky bacillus. It has been proposed to call this disease "Paratyphoid" or "Paracoloid" fever. Brion and Kayser[7] record one such case in full. The colon bacillus was recovered during life from the spots, the urine, and the blood; and the serum of the patient had an agglutinating action for cultures of this bacillus, and also for cultures of a variety of B. coli obtained from Schottmüller's laboratory. The infecting organism differed from the bacillus coli commune in that it did not clot milk or form indol. Grünbaum[8] prefers to call this condition "typhocoloid" fever. It is, he believes, caused by a specific bacillus belonging to the enteritidis group, a bacillus which has the power of fermenting glucose but not lactose. He suggests that all cases of enteric fever which fail to give the Widal reaction should be investigated with regard to the agglutinating property of the serum on members of the intermediate group; that the blood, urine, and fæces should be bateriologically examined, that blood counts should be made, and the diazo reaction of the urine tested. Authentic cases of paracolon infection are rare. Strong[9] reports one from Santa Cruz. The attack differed in no way from true enteric fever. At the post-mortem examination the spleen was enlarged, the mesenteric lymphatics swollen and hæmorrhagic, but there were no intestinal lesions. Cultures from the spleen showed an organism of the coli type, but which did not ferment lactose or form indol. This organism was pathogenic for mice. Strong mentions other cases of paracoloid fever such as those recorded by Widal, Gwyn and Cushing. The two last-named observers isolated their bacilli from the peripheral circulation, and thus established another difference between it and the B. coli which is seldom found outside the abdomen. The *American Journal of Medical Sciences* for August 1902 devotes considerable space to the elucidation of the problem of paratyphoid fever. Johnston reports four new cases. All clinically resembled enteric fever, but were not capable of aggluntinating cultures of B. typhosus. Diarrhœa and termination by crisis are common, myositis and purulent arthritis have been recorded as complications. Intestinal ulceration is rare, and the disease is seldom fatal. Hewlett, in the same magazine, reports a case. He isolated a variety of colon bacillus which, although it clumped typhoid bacilli with a dilution of 1 in 10, yet was far more potent with cultures of Cushing's and Gwynn's paracolon bacilli, which were well clumped in dilutions 1 in 100. In a third article, Longscope describes a case that ended fatally. The spleen was large but no intestinal lesions were discovered. The paracolon bacillus was isolated from the blood, lung, liver and spleen. Widal's reaction with cultures of B. typhosus was negative. In a second case that recovered, the same bacillus was found, and this and the organism from the first case gave positive agglutinating reactions with Cushing's and Gwynn's bacilli. Longscope is of opinion that many of the cases of so-called enteric fever without intestinal lesions are in reality cases of paratyphoid fever, and that in the future the two diseases must be clinically and bacteriologically distinguished. Supposing that the patient were suffering from infection by both the paracolon and the typhoid bacillus, Castellani[10] has shown that the serum would then show agglutinating properties for both organisms, with an intensity unaltered in any respect. Even if the second infection followed the first by a short interval, the two agglutination effects would both coexist. The serum of an animal immunised against two organisms, *a* and *b*, loses when treated with cultures of (*a*) its agglutinating powers for this, but not in any degree for (*b*), and *vice versâ*; treated with both it loses its powers for both. Thus, if when examining the blood of a typhoid patient we find that it agglutinates both cultures of the typhoid and coli bacilli, then the serum should be saturated with B. typhosus, and if then the serum agglutinates cultures of B. coli only, the infection is shown to be a mixed one.

[1] Jour. of Hyg., October 1902. [2] Münch Med. Woch., April 30, 1901. [3] Jour. Path. and Bact, September 1902. [4] Rep. Liverpool Sch. of Tropical Med. [5] Brit. Med. Jour., November 8, 1902. [6] Amer. Med., May 18, 1901. [7] Münch. Med. Woch., April 15, 1902. [8] Brit. Med. Jour., September 20, 1902. [9] Johns Hopkins Hosp. Bull., May 1902. [10] Epit. 307, Brit. Med. Jour., November 15, 1902.

